# Data-driven characterization of traumatic brain injury severity from clinical, neuroimaging, and blood-based indicators

**DOI:** 10.21203/rs.3.rs-3954157/v1

**Published:** 2024-02-16

**Authors:** Lindsay Nelson, Brooke Magnus, John Yue, Steve Balsis, Christopher Patrick, Nancy Temkin, Ramon Diaz-Arrastia, Goeffrey Manley

**Affiliations:** Medical College of Wisconsin; Boston College; UC San Francisco; University of Massachusetts Lowell; Florida State University; University of Washington; Univesrity of Pennsylvania; University of California San Francisco

## Abstract

The conventional clinical approach to characterizing traumatic brain injuries (TBIs) as mild, moderate, or severe using the Glasgow Coma Scale (GCS) total score has well-known limitations, prompting calls for more sophisticated strategies to characterize TBI. Here, we use item response theory (IRT) to develop a novel method for quantifying TBI severity that incorporates neuroimaging and blood-based biomarkers along with clinical measures. Within the multicenter Transforming Research and Clinical Knowledge in TBI (TRACK-TBI) study sample (N = 2545), we show that a set of 23 clinical, head computed tomography (CT), and blood-based biomarker variables familiar to clinicians and researchers index a common latent continuum of TBI severity. We illustrate how IRT can be used to identify the relative value of these features to estimate an individual’s position along the TBI severity continuum. Finally, we show that TBI severity scores generated using this novel IRT-based method incrementally predict functional outcome over classic clinical (mild, moderate, severe) or International Mission for Prognosis and Analysis of Clinical Trials in TBI (IMPACT) classification methods. Our findings directly inform ongoing international efforts to refine and deploy new pragmatic, empirically-supported strategies for characterizing TBI, while illustrating a strategy that may be useful to evolve staging systems for other diseases.

## Introduction

Soon after the advent of the Glasgow Coma Scale (GCS)^1^—the first widely propagated method for estimating traumatic brain injury (TBI) severity—clinicians and researchers began classifying injuries into the broad categories of mild (GCS 13–15), moderate (GCS 9–12), or severe (GCS 3–8) TBI^2–5^. This practice began in the early 1980s as a way to describe TBI subpopulations and rapidly became commonplace^3,4^, with later approaches evolving to additionally consider indicators such as duration of posttraumatic amnesia (PTA), duration of loss of consciousness (LOC), and presence versus absence of acute intracranial findings on clinical neuroimaging (generally computed tomography [CT]) scans; Table 1)^6–8^.

Although the GCS-based staging convention addresses clinical and research needs to characterize and communicate about severity, it has been criticized for its lack of nuance and insensitivity to the heterogenous pathologies of TBI^2,9^. The 3-category GCS approach, for example, labels over 90% of TBIs “mild”^10,11^, which can be misleading given the varied, sometimes poor outcomes of GCS 13–15 TBI^9,12,13^. Besides loss of information in categorizing the GCS by total scores,^2^ TBI severity classi cation approaches rely on clinical signs of altered consciousness that may be confounded by non-TBI factors common in trauma patients, such as alcohol/substance intoxication, use of sedatives and analgesics, and extracranial injuries^2,9^. Systems that incorporate objective brain injury biomarkers—i.e., binary ratings of head CT findings—conflate neuroradiologic findings with widely disparate, or even opposing, effects on long-term outcome. In particular, CT findings are considered “positive” for acute intracranial injury due to epidural hematomas (EDH), a neutral or positive prognostic indicator, as well as imaging findings associated with poor long-term outcomes (e.g., subarachnoid hemorrhage [SAH], subdural hematoma [SDH])^14,15^.

Despite recognition of a need to evolve TBI severity grading systems, progress has been limited by a lack of large, well-characterized TBI samples for developing new approaches and a lack of validated, objective biomarkers that can be used clinically to better detect underlying pathophysiology. Additionally, because TBI severity is reflectedin indicators across measurement domains (e.g., clinical, neuroimaging, blood-based markers), tools are needed that can empirically position diverse indicators along the underlying continuum of TBI severity. Fortunately, recent large-scale prospective TBI studies provide invaluable data to cultivate new strategies for characterizing TBI severity. Using the large prospective Transforming Research and Clinical Knowledge in TBI (TRACK-TBI) sample of United States (U.S.) level 1 trauma center patients, we developed a novel data-driven approach to characterize the broader spectrum of TBI severity.

We used item response theory (IRT) to model the continuum of TBI from diverse clinical signs and objective injury-related biomarkers. IRT is a statistical framework suited to identify an individual’s position along a continuum using indicators from differing measurement domains. Following recommendations that new TBI classification systems be pragmatic^16^, analyses focused on classifying TBI severity using variables widely available in the acute care setting or on the near horizon of clinical translation (blood-based biomarkers). In particular, the individual GCS components (eye, motor, verbal), LOC, PTA, and specific head CT findings (e.g., contusion, SDH) were incorporated into models with more granularity than current grading systems, to enable clearer differentiation among patients and empirically determine their location on the severity spectrum. Second, we incorporated several blood-based biomarkers to address calls for incorporating more biological markers into TBI severity grading^17^. Their inclusion was justified by the near-term feasibility of employing them clinically (e.g., two markers were already FDA- and EMA-approved for decisions about neuroimaging and a third was included in Scandanavian guidelines for managing GCS 14–15 TBI)^18–20^. Establishing the relationship between clinically relevant signs of TBI severity may corroborate what is known from clinical experience and studies of individual signs. In establishing that these clinical signs and biomarkers of TBI reflect a single underlying dimension of severity and locating them on that continuum, this study can advance understanding of the spectrum of severity while offering a quantitative tool for further developing and refining practical TBI severity grading approaches.

## Methods

### Study Design and Participants

The TRACK-TBI study is a prospective observational cohort study of 2545 TBI-diagnosed participants aged ≥ 17 years from 18 U.S. level 1 trauma centers, enrolled between 2014–2018, all of whom were included in the current analysis (Table 2). Ethical approval was obtained at each enrolling site. Inclusion criteria were: enrollment within 24 hours of injury, CT scan ordered for clinical care, documentation of TBI consistent with the American Congress of Rehabilitation Medicine definition (i.e., head trauma resulting in neuroimaging structural brain injury and/or evidence of alteration of consciousness), and adequate visual acuity and hearing to complete outcome examinations. Exclusion criteria were being pregnant, in police custody, or on psychiatric hold; history of debilitating mental or neurological disorders; non-English-and non-Spanish-speaking; penetrating TBI; non-survivable (moribund) trauma; severe polytrauma or medical comorbidities (e.g., end-stage cancer) that would interfere with follow-up and outcome assessment; and being in an interventional trial.

### Procedures

#### TBI severity indicators.

Admission GCS scores were extracted from medical records and coded separately for the eye (range 1–4), verbal (1–5), and motor (1–6) component scores. Untestable GCS codes were treated as missing to allow for GCS to be treated as an ordinal variable. Duration of LOC and PTA were collected from medical records and/or participant interviews and coded in categories consistent with the National Institute of Neurological Disorders and Stroke TBI Common Data Elements (CDE)^21^.

Head CT scans performed on admission for clinical purposes were sent to a central imaging repository (Laboratory of Neuro Imaging, Los Angeles, CA, USA) and assessed by one board-certified neuroradiologist for findings consistent with the TBI CDE for Radiologic Imaging^22^. Binary present/absent codes were used for each imaging finding associated with acute head trauma (SAH, acute SDH, etc.).

Blood samples were collected in the hospital within 24 hours of injury, were processed, aliquoted, and stored in a freezer within 2 hours of collection (for biospecimen collection and processing procedures, see: https://tracktbi.ucsf.edu/researchers). Analyses used data for the core set of biomarkers acquired for the full study sample: glial fibrillary acidic protein (GFAP), ubiquitin C-terminal hydrolase (UCH-L1), high-sensitivity C-reactive protein (hsCRP), S100 calcium binding protein B (S100B), and neuron-specific enolase (NSE). GFAP and UCH-L1 were expected to be most informative given their higher specificity to TBI and robust associations with other indicator variables, especially head CT lesions^23–26^. Coded blood samples were shipped from the study’s central repository to site laboratories and analyzed blinded to any clinical information. Plasma samples were analyzed for GFAP and UCH-L1 at Abbott Laboratories (Abbott Park, IL, USA) on either the company’s prototype point-of-care iSTAT Alinity System or the prototype core lab ARCHITECT platform. The measures were highly correlated and converted for analysis to iSTAT equivalent units for analysis.^27^ Analysis of hsCRP was carried out on serum samples by a laboratory at the University College of Dublin using the Abbott ARCHITECT c8000, MULTIGENT CRP Vario assay using the high-sensitivity method (CRP16).^28^ Analysis of S100B was conducted by a laboratory at the University College of Dublin using an electrochemiluminescence immunoassay (Elecsys^®^ S100B; Roche Diagnostics, Penzberg, Germany) on an automated Cobas^®^ system from Roche. Serum samples were thawed in batches at room temperature and centrifuged at 10,000 rcf for 10 min at 4°C prior to testing in duplicate. This assay is the trademarked assay used clinically in Europe for S100B (LoD: <0.005 ug/L; LoQ: not available per package insert; CV: intermediate precision of 20%), which was optimized for serum.^29^ Details regarding the analysis of NSE are available in a prior publication.^30^

#### Functional outcome.

We evaluated incremental validity of our novel IRT-based TBI severity score for predicting functional outcome, as reflected by the Glasgow Outcome Scale-Extended (GOSE), over/above traditional classifications based on GCS total scores. The GOSE is an ordinal measure of global functional outcome that assigns one of 8 scores: 1 = Death; 2 = Vegetative State; 3 = Lower Severe Disability; 4 = Upper Severe Disability; 5 = Lower Moderate Disability; 6 = Upper Moderate Disability; 7 = Lower Good Recovery; 8 = Upper Good Recovery. Two GOSE scores were derived from structured interviews with patients and informants at 2 weeks and 6 months post-injury^31^—a GOSE-ALL score reflecting the overall change in functional independence due to all injury (TBI and extracranial) and a GOSE-TBI score, indicating the change in independence resulting solely from the TBI. Scores were dichotomized for analysis as death (GOSE = 1), unfavorable outcome (GOSE < 5), and incomplete recovery (GOSE < 8).

### Statistical Analysis

Statistical analyses were conducted using R v.4.3.1^32^, apart from initial factor analytic modeling, which was performed in Mplus v.8^33^. IRT analyses were performed using the “mirt” package in R^34^. Descriptive statistics (frequencies/percentages; means/standard deviations) were also computed. We performed exploratory factor analysis (EFA) using diagonally weighted least squares estimation (WLSMV) to evaluate the key assumption underlying unidimensional IRT modeling, that the set of 23 TBI indicators (5 clinical signs, 13 acute head CT findings, and 5 blood-based biomarkers) indexed a single underlying dimension of TBI severity. Sufficient unidimensionality for IRT modeling was defined a *priori* as an EFA first-to-second eigenvalue ratio > 4, and model fit statistics as follows: root mean square error of approximation (RMSEA) < 0.08, comparative fit index (CFI) > 0.90, and Tucker-Lewis Index (TLI) > 0.90^35–38^.

We then fit a 2-parameter/graded response hybrid logistic unidimensional IRT model to the 23 indicators, which assumes a continuous latent dimension underlying the indicators and accommodates both binary items (presence/absence of each CT feature) and ordinal items (LOC and PTA duration, GCS scores). Continuous blood-based biomarker variables were categorized into 6–11 equally sized groups and treated as ordinal items in the IRT model. The model estimates two parameters per item, discrimination (*a*_*j*_) and one or more threshold parameters (*b*_*j*_). *Threshold* (*or difficulty*) reflects the location on the TBI severityaj continuum where a respondent has, for a binary indicator, a 0.5 probability of the indicator or, for a polytomous ordinal item, a 0.5 probability of displaying that category or a more severe one. *Discrimination* reflects the strength of the relationship between the item and the latent dimension; more discriminating items distinguish better between individuals who differ in TBI severity, especially at the threshold/di culty level of the item.^38^ Analyses displayed the overall precision of each TBI indicator and their combined performance in a metric called *information*, which aggregates diffculty and discrimination and reflects the inverse of the standard error of measurement around estimates of the latent variable (TBI severity) across the continuum of severity.

The model yielded IRT-based TBI severity scores for each participant, which were submitted to further analyses to explore their potential validity and utility. We generated scatterplots and histograms to display the association between the novel severity score and traditional TBI classifications (e.g., mild, moderate, severe); computed Spearman correlations to compare associations of GCS scores and novel TBI severity scores with functional (GOSE) outcomes; and fit separate sequential binary logistic regression models to examine the independent predictive value of TBI severity scores over/above (i) traditional GCS-based mild, moderate, and severe TBI classification, and (ii) International Mission for Prognosis and Analysis of Clinical Trials in TBI (IMPACT) scores^14^.

IMPACT scores, developed from readily available acute injury indicators in patients with GCS < 13, are well-established to prognosticate functional (GOSE) outcome in this TBI subpopulation^14,39^. Models were run separately using the IMPACT Core model (comprising age, GCS motor score, and pupil reactivity) and the IMPACT Extended model (which adds to the Core model hypotension, hypoxia, and select head CT features), each of which produces separate scores for predicting mortality and unfavorable outcome (for details, see: http://www.tbi-impact.org/?p=impact/calc.)

To evaluate the incremental validity of novel IRT-based TBI severity scores over the GCS-based severity group (mild, moderate, severe; among the full GCS 3–15 sample) or IMPACT prognostic score (among the GCS < 13 subsample), we computed the percentage of variability in GOSE outcomes associated with adding novel TBI severity scores to each model (estimated by Nagelkerke R^2^)^40^.

## Results

### Fit of unidimensional IRT model

The EFA of the 23 acute TBI indicators supported proceeding with unidimensional IRT modeling and indicated that these diverse clinical signs, head CT findings, and blood-based biomarkers reflect a single common dimension, which we refer to here as TBI severity. Specifically, the first-to-second eigenvalue ratio was above 4 (12.55/2.15 = 5.8) and model fit met the aforementioned criteria for sufficient unidimensionality (RMSEA .079, CFI .962, TLI .958). The scree plot and factor loadings for the EFA model appear in the online Extended Data (**eFigure 1; eTable 1**).

### Characteristics of the IRT model

**Figure 1:**
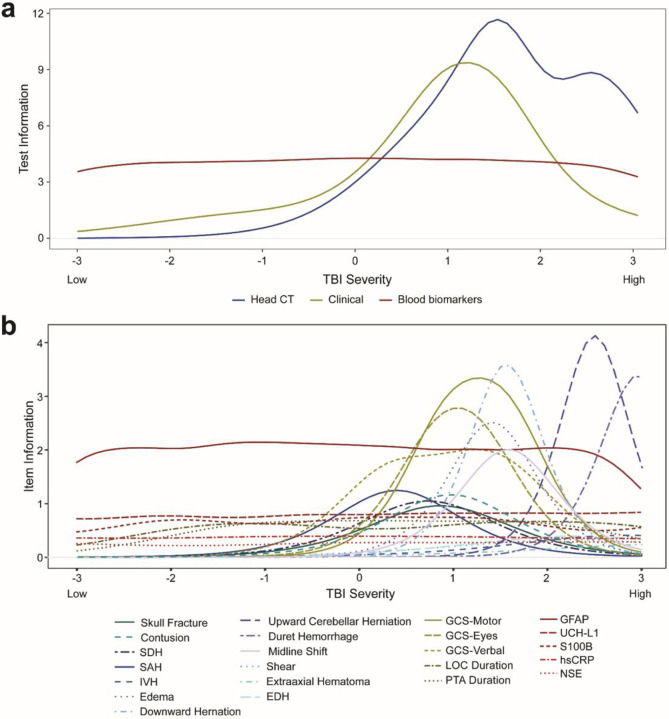
Test information curves for each measurement domain. Test information reflects the sum of information across all indicators (items) in each measurement domain. Higher information reflects greater measurement precision in characterizing and distinguishing persons at a given level of TBI severity.

**Figure 2:**
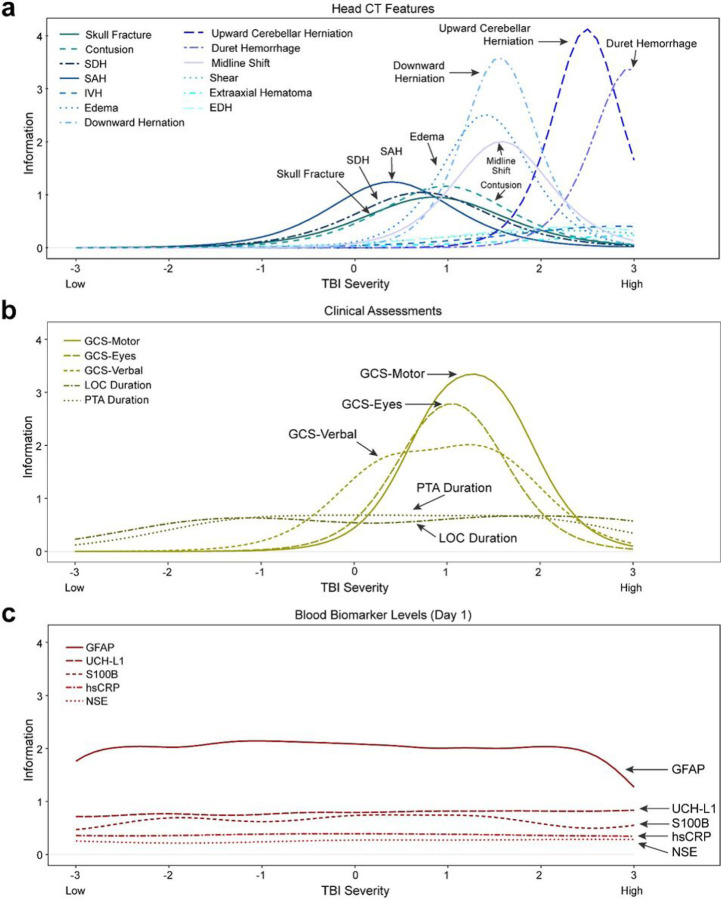
Item information curves, stratified by measurement domain of the TBI severity indicators.

IRT model parameters are provided in **eTable 2** (online Extended Data) which, in combination, can be visualized as test (Fig. 1a) and item (Fig. 1b; Fig. 2) information curves, where test information curves sum the information from the items that fall into each respective measurement domain (clinical signs, head CT findings, blood-based biomarker levels). Positive head CT findings indexed the highest end of the severity spectrum. Clinical signs of GCS domain scores, LOC duration, and PTA duration best measured the moderate-severe end of the severity spectrum, although they also contributed more information than head CT features in the lower half of the spectrum. The added information provided by clinical features at lower levels of severity was accounted for by contributions of LOC and PTA duration (**eFigure 2**).

Item information curves are provided in Fig. 1b (not stratified, to facilitate comparisons across domain) and Fig. 2 (stratified by measurement domain to facilitate readability). The item information curves convey both where the individual features/items fall along the injury spectrum, and which features are better- or worse-performing. For example, while all GCS domains indexed a similar level of severity, the motor score displayed the highest information (precision). Head CT features varied markedly in the severity of injury they indexed. For example, Duret hemorrhage—a brainstem hemorrhage associated with cerebral herniation—provided high information at the right-most portion of the spectrum, indicating that it operates as a sensitive index of the most severe injuries. Evidence of herniation, edema, and midline shift contributed information about mid-range TBI severity. Contusion, SAH, and SDH also provided moderate information about this range of the severity spectrum. Other features (e.g., EDH) provided little information (i.e., little ability to help differentiate individuals varying in severity). Skull fracture, although not technically a brain-injury-specific finding, fit within the model and indexed the latent spectrum similarly to SDH and contusion.

Finally, the relatively flat line of the blood-based biomarker test information function (Fig. 1a) indicates that blood-based biomarkers contributed to indexing the entire spectrum of TBI severity, which appeared driven by high performance of GFAP in particular (Fig. 1b, Fig. 2). In the lower half of the spectrum (< 0), blood-based biomarkers contributed to a marked increase in measurement precision as compared with clinical and CT features.

### Associations between GCS-based TBI severity groups, novel IRT-based severity scores, and functional outcomes

**Figure 3:**
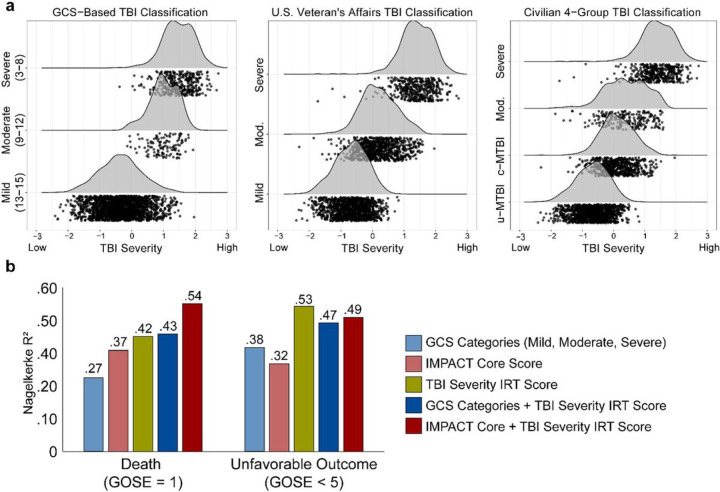
Association between novel item response theory-based traumatic brain injury (TBI) severity scores, other TBI severity classification schemes, and functional outcome. **(a)** Histograms and scatterplots of item response theory TBI severity score versus traditional TBI severity classification schemes. Scatterplot points (individual subjects) are lagged in the direction of the y-axis to facilitate visualization of the number of points along the x-axis. **(b)** Model Nagelkerke R2 for models predicting death and unfavorable outcome at 6 months post-injury from traditional 3-level GCS-based mild, moderate, and severe categories, IMPACT Core model scores, and IRT-based TBI severity scores, as well as multivariable models investigating the incremental predictive value of IRT-based TBI severity scores in combination with the other variables. *Abbreviations*: c-mTBI, complicated mild TBI; GCS, Glasgow Coma Scale score; IMPACT, International Mission for Prognosis and Analysis of Clinical Trials in TBI; Mod, Moderate; u-mTBI, uncomplicated mild TBI

Model-based estimates of TBI severity (*IRT* scores), scaled in z-score units, were derived for each participant for use in subsequent analyses. Leveraging the information contained within the 23 items produced a score with much more granularity than classic mild, moderate, and severe TBI categories (see Fig. 3a). The monotonic relationship observed between the IRT scores and traditional categories supports the validity of the model.

**eTable 3** shows Spearman correlations between IRT scores, GCS scores, and functional outcome, stratified by GCS-based mild, moderate, and severe categories. IRT scores correlated robustly with GOSE outcomes, particularly for the GCS 9–12 and GCS 3–8 strata. Associations were modest within the GCS 13–15 stratum but nevertheless more robust than continuous GCS scores.

Finally, logistic regression models demonstrated that IRT-based TBI severity scores incrementally predicted all 6-month GOSE outcomes evaluated (i.e., death, unfavorable outcome, and incomplete recovery relative to GCS-based classifications [Table 3] and death and unfavorable outcome relative to IMPACT Core and Extended scores [Table 3]). Gains in predictive power were especially strong when predicting death and unfavorable outcome. For example, even after accounting for GCS-based classification, each one-point increase in IRT-based TBI severity scores was associated with 12.32 (95% CI, 7.46, 21.20) times the odds of death and 5.56 (95% CI, 3.94, 7.97) times the odds of unfavorable outcome (i.e., Nagelkerke R^2^s increased from .27–43 and .38–.47, respectively, with addition of IRT-based scores to the model). Similar predictive gains were evident when IRT-based TBI severity scores were added to models alongside IMPACT scores. For example, the increase in odds of death (OR) for every one-point increase in the acute IRT severity score, after adjusting for IMPACT score, was 26.33 (95% CI 9.85, 79.77) for the IMPACT Core model and 11.95 (95% CI, 3.32, 50.03) for the IMPACT Extended model. The corresponding ORs predicting unfavorable outcome were 12.53 (95% CI, 9.01, 28.02) for the IMPACT Core model and 12.18 (95% CI, 4.27, 38.61) for the IMPACT Extended model. For reference, **eTable 4** provides regression model output predicting GOSE outcomes from the IRT severity score alone. Nagelkerke R^2^ values for all models are illustrated in Fig. 3b.

## Discussion

In a large (N = 2545) sample of level 1 trauma center patients aged 17 years and older with GCS 3–15 TBI, we used IRT to model the continuum of TBI severity from 23 clinical, head CT, and blood-based biomarker features assessable soon after injury. Our findings were fourfold. First, finding good fit of a 1-factor model provided empirical support for the widespread assumptions of a dimension of TBI severity that can be indexed, in part, by the individual GCS components, duration of altered consciousness (LOC, PTA), and specific clinical head CT findings. Second, IRT information curves provided a novel view of the level of TBI severity indexed by each indicator and the relative ability of the indicators to characterize individuals’ positions along the severity continuum. Third, we demonstrated that blood-based biomarkers collected within 24 hours of injury, especially GFAP, contribute to indexing the entire continuum of injury. Finally, we demonstrated the validity of novel IRT-based TBI severity scores by showing that these related as expected with traditional GCS-based severity categories and incrementally improved prediction of functional outcome beyond GCS-based severity categories and IMPACT scores.

This study fills a need to integrate data from multiple measurement domains to develop evidence-based, pragmatic TBI severity grading approaches. It also provides an initial demonstration of the utility of IRT for addressing longstanding challenges in the classification and staging of TBI, a problem that could be further addressed through additional applications of this tool–for example, by investigating whether adding other acute severity indicators (e.g., pupillary reactivity, hypotension, hypoxia)^14^, or variables reflecting patients’ evolving clinical course, improves characterization of different levels of TBI severity^41^. IRT also provides a quantitative framework for developing simpler classification systems, through examination of effects on model precision of omitting redundant or lower-performing variables, to balance goals of precision and parsimony.

Our results contribute to growing evidence that incorporating blood-based biomarkers may improve characterization of TBI severity^41^. For example, our findings suggest that GFAP, currently FDA-cleared to assist in the decision to pursue clinical neuroimaging^19^, may also indicate level of TBI severity–by contributing to differentiation especially (relative to clinical and CT variables) in the lower half of the severity spectrum, where most individuals show no other objective (e.g., CT) biomarkers of injury. Two other widely used TBI biomarkers, UCH-L1 and S100B, improved characterization of severity but less so than GFAP. However, we caution against drawing firm conclusions about the relative performance of biomarkers from this study alone, as the markers we used vary widely in their half-lives, and TRACK-TBI blood samples were collected at a more optimal timepoint for detecting GFAP.

A strength of this study was that IRT analyses included the full TRACK-TBI sample, a feature enabled by limited missingness on most variables and the use of full-information methods for IRT parameter estimation. This, alongside the diverse 18-site sample, theoretically maximizes generalizability of the model to U.S. level 1 trauma centers. Moreover, in emphasizing the most widely used variables in current TBI severity classification approaches (e.g., GCS, LOC/PTA, head CT findings), our findings directly inform current classification approaches.

We recognize several limitations. Additional investigations will inform the utility of adding other candidate severity indicators to the model. Future studies may also verify model fit to important subgroups. Further, measurements of some variables may not have been optimal. Blood for the current analyses was sampled at one timepoint within 24 hours of injury (12 hours or later for many samples), often missing the early peak of UCH-L1 and S100B and preceding the peak of hsCRP. The relative information provided by the blood-based biomarkers should be interpreted with this limitation in mind. Additionally, assessments of LOC and PTA duration were not standardized, and PTA was not assessed through serial formal assessment. While this underscores their value for measuring TBI severity, it also raises the possibility of that these clinical signs could be more informative if assessed under more controlled conditions.

It is worth noting that there are a multitude of quantitative approaches to staging, or grading, disease severity. For example, IMPACT scores were developed to predict functional outcome and, in turn, provide an early estimate of TBI severity. In contrast, our IRT-based scores were developed without regard to outcomes, instead relying on the assumption of a latent dimension underlying observed associations among TBI indicators and modeling that dimension using the observed indicators in a manner that best reproduces covariation among them. Of note, the finding that the IRT-based severity scores incrementally predicted functional outcomes when combined with IMPACT scores suggests that IRT methods can complement these validated approaches aimed at maximizing prognostication in conceptualizing and quantifying TBI severity.

Taken together, our study provides novel, clinically interpretable findings regarding the manner in which diverse clinical, neuroimaging, and blood-based biomarker variables contribute to indexing the continuum of TBI severity. The IRT approach is sufficiently flexible to enable future integration of other measures related to physiological brain injury and outcome, such as lesion laterality and location, post-acute clinical decline and radiographic progression of intracranial injury, markers of secondary injury, and potentially psychosocial or environmental factors. IRT methodology can help delineate the contributions of diverse variables to a composite, comprehensive TBI severity classification, while improving the understanding of the varied premorbid and injury factors that have limited traditional approaches to severity classification. A number of these indicators are currently under curation in TRACK-TBI and could be included in follow-up studies. In summary, this study demonstrates the potential utility of IRT to contribute to developing and validating practical, empirically-supported TBI severity grading systems.

## Figures and Tables

**Table 1:** Three commonly used TBI classification systems based on acute injury characteristics.

GCS-based	Mild		Moderate	Severe
	GCS 13–15		GCS 9–12	GCS 3–8
**VA 3-group**	**Mild**		**Moderate**	**Severe**
	GCS 13–15		GCS 9–12	GCS3–8
	LOC < 30 minutes		LOC >30 min. & < 24 hr.	LOC >24 hr.
	PTA < 24 hours		PTA > 24 hr. & < 7 d	PTA >7 d.
	AMS up to 24 hr.		AMS >24 hr.	AMS >24 h.
	CT-		CT- or CT+	CT- or CT+
**Civilian 4- group**	**Uncomplicated Mild**	**Complicated Mild**	**Moderate**	**Severe**
	GCS 13–15	GCS 13–15	GCS 9–12	GCS3–8
	LOC < 30 minutes	LOC < 30 minutes	30 min. < LOC <24 hr.	LOC >24 hr.
	PTA < 24 hours	PTA< 24 hours	24 hr < PTA < 7 d	PTA >7 d.
	CT-	CT+		

*Note*. AMS, alteration of consciousness/mental status; CT, head computed tomography scan (CT + reflects the presence of acute intracranial findings, CT- reflects that acute intracranial findings are absent or the test was not performed); GCS, Glasgow Coma Scale score; LOC, loss of consciousness; PTA, posttraumatic amnesia

1 If a patient meets criteria in more than one category of severity, the higher severity level is assigned.

**Table 2:** Sample characteristics (*N* = 2545)

	*n(%)* or *M(SD)*
Demographics	
Age, years	41.3 (17.5)
Sex (male)	1762 (69.2%)
Race	
Asian	93 (3.7%)
Black	406 (16.0%)
White	1949 (76.6%)
Other/unknown	97 (3.8%)
Ethnicity	
Hispanic	516 (20.3%)
Non-Hispanic	1990 (78.2%)
Unknown	39 (1.5%)
Education, years	13.32 (2.87)
Health insurance	
Medicaid/uninsured	789 (31.0%)
Other insurance	1571 (61.7%)
Unknown	185 (7.3%)
Injury characteristics	
Admission GCS score, M (SD)^1^	13.0 (3.8)
3–8, n (%)	360 (14.5%)
10–12, n (%)	123 (5.0%)
13–15, n (%)	1995 (80.5%)
Positive head CT^1^	1162 (47.7%)
Cause of injury	
Motor vehicle/traffic crash	1456 (57.2%)
Fall	673 (26.4%)
Assault/violence	168 (6.6%)
Other/unknown	248 (9.7%)
Highest level of care	
Emergency department	530 (20.8%)
Inpatient floor	870 (34.2%)
Intensive care unit	1145 (45.0%)
Loss of consciousness duration^1^	
None	282 (15.8%)
< 1 min.	336 (18.8%)
1–29 min	828 (46.4%)
30–59 min	61 (3.4%)
1–24 h	114 (6.4%)
>24 h	94 (5.3%)
>7 days	70 (3.9%)
Posttraumatic amnesia duration^1^	
None	416 (23.8%)
< 1 min.	98 (5.6%)
1–29 min	494 (28.2%)
30–59 min	138 (28.2%)
1–24 h	363 (20.7%
>24 h	121 (6.9%)
>7 days	121 (6.9%)

*Note*. Sample reflects all TRACK-TBI study participants who had traumatic brain injury and were at least 17 years old.

1 n = 77 missing Admission GCS; n = 110 missing Head CT outcome; n = 760 missing loss of consciousness duration; n = 794 missing posttraumatic amnesia duration

**Table 3:** Incremental predictive value of acute TBI severity IRT scores as compared to GCS-based classification of mild, moderate, or severe TBI (top) and IMPACT scores (bottom) for predicting 6-month functional outcomes.

Models Adding IRT-Based TBI Severity Scores to Traditional GCS-Based 3-Category Severity										
		Step 1					Step 2			
Death		B (SE)		OR (95% CI)	P		B (SE)	OR (95% CI)		P
GCS					< .001					.152
GCS 9–12 vs. GCS 13–15		1.92 (0.41)		6.84 (2.92, 14.75)	< .001		−0.76 (0.47)	0.47 (0.18, 1.14)		.105
GCS 3–8 vs. GCS 13–15		2.97 (0.24)		19.41 (12.24, 31.77)	< .001		−0.71 (0.39)	0.49 (0.23, 1.07)		.070
TBI Severity IRT score							2.51 (0.26)	12.32 (7.46, 21.10)		< .001
Model fit - overall		χ^2^(2) = 182.93 (p .001)					χ^2^(3) = 301.89 (p < .001)			
Model fit - step 2							χ^2^(1) = 118.96 (p < .001)			
Nagelkerke R^2^		.27					.43			
Unfavorable outcome		B (SE)		OR (95% CI)	P		B (SE)	OR (95% CI)		P
GCS					< .001					< .001
GCS 9–12 vs. GCS 13–15		2.62 (0.28)		13.70 (7.93, 23.48)	< .001		0.65 (0.33)	1.91 (1.00, 3.65)		.048
GCS 3–8 vs. GCS 13–15		3.24 (0.18)		25.42 (17.82, 36.76)	< .001		0.62 (0.29)	1.86 (1.05, 3.32)		.034
TBI Severity IRT score							1.72 (0.18)	5.56 (3.94, 7.97)		< .001
Model fit - overall		χ^2^(2) = 389.78 (p < .001)					χ^2^(3) = 529.14 (p < .001)			
Model fit - step 2							χ^2^(1) = 106.05 (p < .001)			
Nagelkerke R^2^		.38					.47			
Incomplete recovery		B (SE)		OR (95% CI)	P		B (SE)	OR (95% CI)		P
GCS					< .001					< .001
GCS 9–12 vs. GCS 13–15		1.24 (0.34)		3.44 (1.75, 6.76)	< .001		0.69 (0.37)	1.99 (1.01, 4.31)		.061
GCS 3–8 vs. GCS 13–15		1.67 (0.22)		5.30 (3.46, 8.12)	< .001		0.96 (0.27)	2.61 (1.56, 4.51)		< .001
TBI Severity IRT score							0.38 (0.08)	1.46 (1.24, 1.74)		< .001
Model fit - overall		χ^2^(2) = 95.39 (p < .001)					χ^2^(3) = 114.99 (p < .001)			
Model fit - step 2							χ^2^(1) = 19.60 (p < .001)			
Nagelkerke R^2^		.08					.10			
Models Adding IRT-Based TBI Severity Scores to IMPACT Scores										
		Step 1					Step 2			
Death		B (SE)		OR (95% CI)	P		B (SE)		OR (95% CI)	P
IMPACT Core score		1.37 (0.18)		3.93 (2.82, 5.73)	< .001		1.07 (0.20)		2.91 (2.00, 4.42)	< .001
Acute IRT severity score							3.27 (0.53)		26.33 (9.85, 79.77)	< .001
Model fit - overall		χ^2^(1) = 89.78 (p < .001)					χ^2^(2) = 144.32 (p < .001)			
Model fit - step 2							χ^2^(1) = 54.54 (p < .001)			
Nagelkerke R^2^		.37					.54			
		B (SE)		OR (95% CI)	P		B (SE)		OR (95% CI)	P
IMPACT Extended score		1.42 (0.22)		4.15 (2.79, 6.59)	< .001		0.95 (0.24)		2.58 (1.64. 4.28)	< .001
TBI Severity IRT score							2.48 (0.69)		11.95 (3.32, 50.03)	< .001
Model fit - overall	χ^2^(1) = 74.45 (p < .001)						χ^2^(2) = 90.10 (p < .001)			
Model fit - step 2							χ^2^(1) = 15.65 (p < .001)			
Nagelkerke R^2^	.42						.50			
Unfavorable outcome	B (SE)		OR (95% CI)			P	B (SE)		OR (95% CI)	P
IMPACT Core score	1.11 (0.14)		3.06 (2.35, 4.09)			< .001	0.86 (0.16)		2.36 (1.76, 3.23)	< .001
TBI Severity IRT score							2.53 (0.39)		12.53 (6.01, 28.02)	< .001
Model fit - overall	χ^2^(1) = 88.02 (p < .001)						χ^2^(2) = 143.20 (p < .001)			
Model fit - step 2							χ^2^(1) = 55.18, (p < .001)			
Nagelkerke R^2^	.32						.49			
	B (SE)		OR (95% CI)			P	B (SE)		OR (95% CI)	P
IMPACT Extended score	1.39 (0.19)		4.01 (2.85, 5.92)			< .001	0.96 (0.21)		2.61 (1.78, 4.00)	< .001
TBI Severity IRT score							2.50 (0.56)		12.18 (4.27, 38.61)	< .001
Model fit - overall	χ^2^(1) = 97.92, (p < .001)						χ^2^(2) = 122.29 (p < .001)			
Model fit - step 2							χ^2^(1) = 24.37 (p < .001)			
Nagelkerke R^2^	.47						.56			

*Note*. Model Ns for models incorporating GCS-based TBI categories were *N* = 1653. Models incorporating IMPACT scores included the subsample who were GCS < 13 and who had all IMPACT indicators available (Model *Ns* = 227 to 316). Outcomes reflect Glasgow Outcome Scale-Extended (GOSE) scores at 6 months post-injury of 1 (death), < 5 (unfavorable outcome), and incomplete recovery (GOSE < 8).
